# Advancing early detection of organ damage and cardiovascular risk prevention: the Suzhou cardiometabolic health study protocol - exploring the role of oral microbiome and metabolic profiling in risk stratification

**DOI:** 10.3389/fendo.2025.1522756

**Published:** 2025-02-12

**Authors:** Mengmeng Zhu, Yiwen Li, Wenting Wang, Longkun Liu, Wenwu Liu, Jiayu Yu, Qian Xu, Jing Cui, Yanfei Liu, Keji Chen, Yue Liu

**Affiliations:** ^1^ National Clinical Research Center for Cardiovascular Diseases of Traditional Chinese Medicine, Xiyuan Hospital of China Academy of Chinese Medical Sciences, Beijing, China; ^2^ Cardiovascular disease center, Suzhou hospital of Traditional Chinese medicine, Xiyuan hospital of China academy of Chinese medical sciences, Suzhou, Jiangsu, China; ^3^ Beijing Key Laboratory of Traditional Chinese Medicine Basic Research on Prevention and Treatment for Major Diseases, Experimental Research Center, China Academy of Chinese Medical Sciences, Beijing, China

**Keywords:** cardiovascular disease, metabolic risk factors, subclinical target organ damage, oral microbiota, risk stratification

## Abstract

**Background:**

Cardiovascular Disease (CVD) is the leading cause of global mortality, with its incidence rate rising year by year due to the prevalence of metabolic diseases. Existing primary and secondary prevention strategies for cardiovascular disease have limitations in identifying some high-risk groups, and 1.5-level prevention aims to achieve more precise intervention by early identification of subclinical target organ damage. This study introduces oral (tongue coating) microbiota as metabolic markers for the first time, in combination with multiple metabolic factors, to explore their potential in assessing subclinical target organ damage and optimizing cardiovascular risk stratification, in order to provide a new path for the early identification and intervention of CVD.

**Methods:**

This study is a prospective cohort study aimed at assessing the association between tongue coating microbiota characteristics and multiple metabolic factors with subclinical target organ damage, and identifying high-risk groups suitable for cardiovascular 1.5-level prevention. The study will be conducted in Suzhou City, Jiangsu Province, China, planning to include 5000-6000 eligible subjects, with inclusion criteria of age ≥18 years, excluding individuals with a history of CVD and other serious diseases. Baseline assessment includes demographic information, lifestyle (including dietary patterns), medical history, physical examination, and collection of tongue coating microbiota samples. Subjects will be followed up every 2 years, with the primary outcome being the first occurrence of coronary heart disease and stroke, and the secondary outcome being subclinical target organ damage.

**Discussion:**

This study focuses on cardiovascular 1.5-level prevention strategy, combining metabolic factors with tongue coating microbiota characteristics, aiming to optimize the risk assessment system for subclinical target organ damage. This approach can not only fill the gap in traditional risk assessment but also provide new ideas for the early identification and intervention of CVD. In the future, the feasibility and effectiveness of this strategy will be verified through multicenter studies, and it is expected to be promoted to a wider medical system, significantly improving the health management level of high-risk groups for CVD.

**Trial registration number:**

http://itmctr.ccebtcm.org.cn, identifier ITMCTR2024000616.

## Graphical Abstract

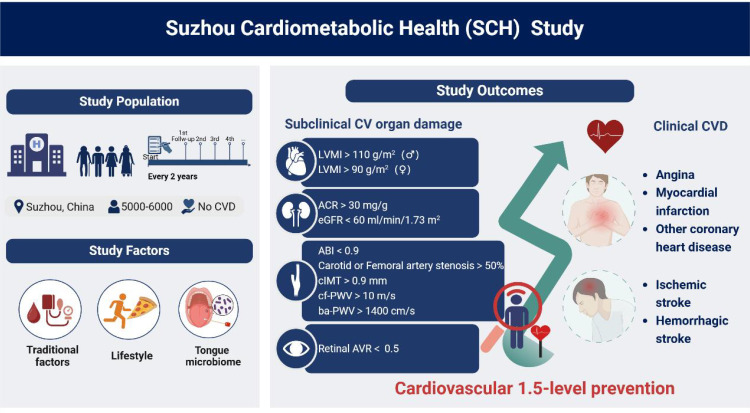

## Introduction

Cardiovascular Disease (CVD) is the leading cause of death and disease burden worldwide (1). With the prevalence of metabolic diseases (such as diabetes, obesity, etc.) (2), the high incidence rate of CVD has become increasingly severe, and the relationship between the two has become closer (3, 4). Although primary and secondary prevention strategies for CVD have been widely applied in clinical practice, they still have significant deficiencies in comprehensively reducing the incidence of CVD (5). The current primary prevention has limited effectiveness in identifying high-risk and elderly populations, leading to many potential beneficiaries not receiving timely intervention, which is difficult to meet the needs of large-scale prevention (6, 7). In terms of secondary prevention, a large number of patients do not receive continuous and comprehensive management support after the first cardiovascular event, resulting in a high recurrence rate of myocardial infarction and stroke (8–10). This indicates that relying solely on existing prevention strategies is difficult to achieve comprehensive management of CVD, and new assessment and management methods are urgently needed to more accurately identify and intervene in potential cardiovascular risks.

In the progression of CVD, many patients have subclinical target organ damage at an early stage, such as left ventricular hypertrophy (LVH), microalbuminuria (MAU), and arteriosclerosis, which usually exist before clinical symptoms appear (11). Early organ damage not only significantly increases the risk of cardiovascular events but also has a profound impact on the long-term prognosis of patients (12, 13). However, the traditional CVD risk assessment system is difficult to effectively capture this period of damage, leading to inaccurate screening of high-risk individuals. Therefore, to fill the gap between primary and secondary prevention, the concept of cardiovascular 1.5-level prevention has emerged. The core of cardiovascular 1.5-level prevention is to identify and manage subclinical target organ damage, and reduce the risk of future cardiovascular events through earlier and more personalized intervention measures (14).

In recent years, with the increase of people with unhealthy lifestyles and the expansion of the base of metabolic abnormal patients, the potential risk factors for CVD have also become more complex (15, 16). Even if some individuals do not show typical metabolic risk factors, they may still face a higher risk of cardiovascular events (17). Therefore, we hope to establish a more comprehensive cardiovascular risk stratification system by integrating the assessment of multiple metabolic factors and subclinical target organ damage, providing a scientific basis for the identification of hidden high-risk groups.

This study innovatively introduces the tongue coating microbiota as a new type of metabolic marker, emphasizing its potential value in cardiovascular risk assessment. In recent years, the connection between the microbiota and systemic metabolic disorders has gradually attracted attention (18), and the composition, microbiota age, and metabolic state of the microbiota are closely related to various metabolic diseases and cardiovascular events (19). Among them, the oral microbiota can affect cardiovascular risk through multiple pathways such as inflammation, immune regulation, and endothelial dysfunction (20, 21), among which the tongue coating microbiota is conveniently sampled, and the characteristics of the tongue coating microbiota can distinguish and even predict the disease status of the human body (22, 23). Based on the above background, this study aims to investigate the novel concept of the oral microbiota, by measuring the diversity, abundance, relative proportion of specific microbiota, and their metabolic functions of the tongue coating microbiota, revealing the characteristics of the tongue coating microbiota in different age groups and metabolic states, and assessing its feasibility as a predictive biomarker for CVD risk.

**Figure 1:**
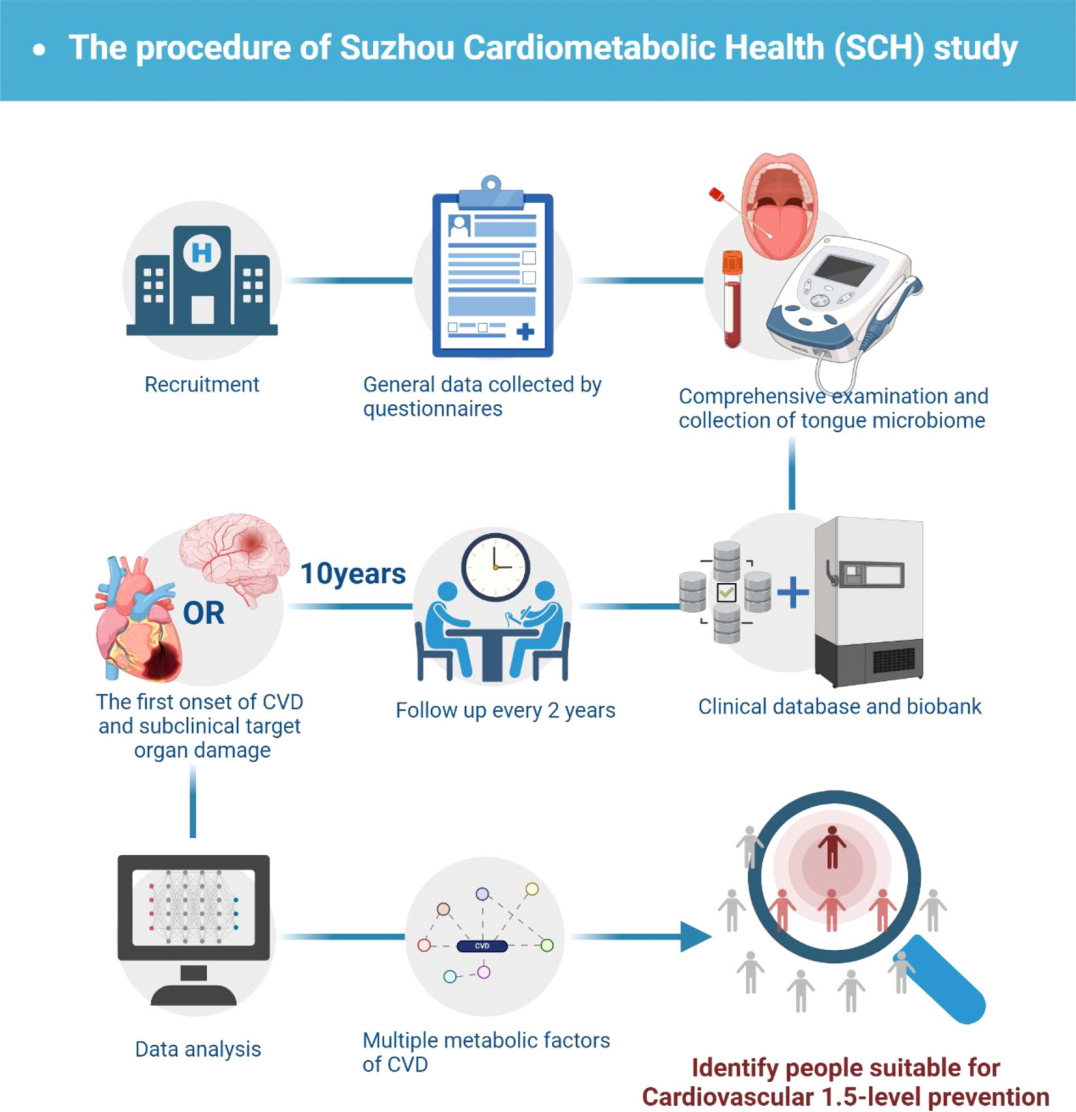
Study flowchart.

To address the above challenges, we have launched the “Suzhou Cardiometabolic Health (SCH) Study” in the Suzhou area of China. This study is a large-scale prospective observational cohort study aimed at integrating tongue coating microbiota characteristics with multiple metabolic factors, systematically assessing their relationship with subclinical target organ damage, and constructing a multi-dimensional risk assessment model to optimize cardiovascular risk stratification. This model is expected to play a key role in the early identification and intervention of high-risk individuals, providing a scientific basis for the implementation of cardiovascular 1.5-level prevention and the formulation of individualized intervention strategies, promoting precise prevention and intervention of CVD (**Figure 1**).

## Methods

### Objectives

To explore the relationship between tongue coating microbiota characteristics and multiple metabolic factors and subclinical target organ damage, to optimize the risk stratification of CVD, and to provide a basis for the selection of 1.5-level prevention targets.

### Study population

Inclusion criteria include (1): age ≥18 years; (2) voluntarily signing an informed consent form (3); capable of long-term follow-up.

Exclusion criteria include: (1) history of CVD, including coronary heart disease, heart failure, stroke, peripheral arterial disease; (2) severe liver and kidney dysfunction; (3) cancer or life expectancy <5 years; (4) pregnant or lactating women; (5) mental or cognitive disorders and other individuals who cannot cooperate; (6) those who have already participated in other clinical studies.

### Recruitment

Recruitment is carried out in Suzhou City, Jiangsu Province, China, by full-time project personnel from Xiyuan Hospital Suzhou Hospital (Suzhou Hospital of Traditional Chinese Medicine). A combination of online and offline recruitment methods will be used to ensure the recruitment of participants in the widest range: (1) promote the study information and recruitment notices through social media platforms; (2) post recruitment posters at Xiyuan Hospital Suzhou Hospital (Suzhou Hospital of Traditional Chinese Medicine) and related community hospitals, detailing the purpose of the study, participation requirements, and contact information; (3) distribute recruitment flyers at resident committees and community activity centers, communicate directly with potential participants, and answer related questions.

### Baseline assessment

**Figure 2:**
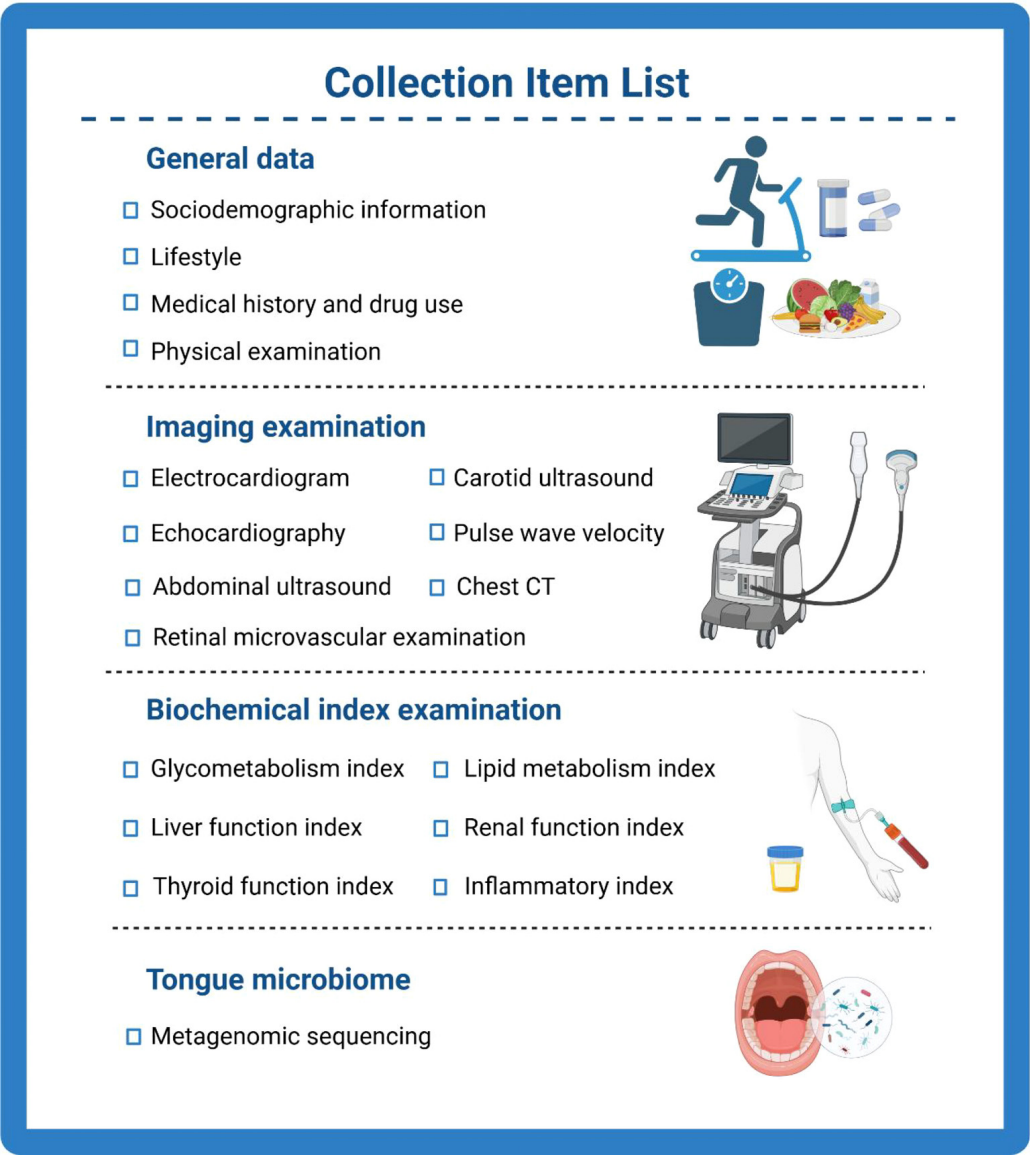
Overview of information collection.

Baseline assessment includes general data collection, physical examination, laboratory tests, imaging examinations, and collection of tongue coating microbiota samples (**Figure 2**). To standardize research methods and procedures, all researchers received training before the start of the study. The specific items are described below.

General data collection:

General data is collected using standardized questionnaires and face-to-face interviews, including socio-demographic information, lifestyle, medical history, and medication use.

Socio-demographic information: including gender, age, race, marital status, education level, occupation, place of residence.

Lifestyle: collecting information on smoking, drinking, physical activity, sleep, and dietary patterns of subjects.

Smoking: divided into never smokers, former smokers, occasional smokers, and current regular smokers, with never smokers referring to those who do not smoke at the time of the baseline interview and have smoked less than 100 cigarettes in their lifetime; former smokers refer to those who have smoked at least 100 cigarettes but have quit smoking for at least 6 months at the time of the baseline interview; occasional smokers refer to those who do not meet the standard of never smokers and have not completely quit smoking for at least 6 months at the time of the baseline interview. Current regular smokers are those who smoke at least one cigarette a day for at least 6 months.

Drinking: divided into never drinkers, former drinkers, occasional drinkers, and current regular drinkers, with never drinkers referring to those who do not drink at the time of the baseline interview and have never had a continuous 6 months of weekly drinking in their lifetime; former drinking history refers to those who have had at least 6 months of weekly drinking of alcoholic beverages in the past but have abstained from alcohol for at least 6 months at the time of the baseline interview; occasional drinking history refers to those who do not meet the standard of never drinkers and have not completely quit drinking for at least 6 months at the time of the baseline interview; current regular drinkers are those who drink at least once a week for at least 6 months. Current regular drinkers are divided into excessive drinkers and non-excessive drinkers, with excessive drinking defined as at least 20g of alcohol per day for women and at least 40g for men.

Physical activity: the International Physical Activity Questionnaire Short Form (IPAQ-SF) is used to survey (24), asking individuals about their physical activities related to work, transportation, housework, and leisure in the past 7 days, as well as the frequency and daily cumulative time of physical activities of different intensities (**Supplementary Table S1**). Sleep is divided into short sleep, moderate sleep, and excessive sleep, corresponding to less than 6 hours per day, 6 to 8 hours per day, and more than 8 hours per day, respectively.

Dietary patterns: a food frequency questionnaire (FFQ) designed according to the dietary characteristics of southern China is used to assess the dietary intake of patients in the past 12 months(**Supplementary Table S2**). The design of this questionnaire refers to the internationally common FFQ content (25, 26) and combines the dietary characteristics of the Jiangnan area in China (27, 28), covering various typical Jiangnan food categories, such as rice, noodles, bean products, freshwater fish, etc., combining the frequency, portion, and cooking methods of food intake to fully capture the daily dietary patterns of the Suzhou area population. In addition, for subjects receiving nutritional treatment guidance, their dietary intervention plans and actual intake will be recorded separately.

Medical history and medication use: investigate the medical history of diabetes, hypertension, etc., and verify through medical or hospital records. Investigate the family history of CVD among patients and their age of onset, and those with CVD in first-degree relatives (men <55 years, women <65 years) are determined to have a family history of early-onset CVD. Collect the self-reported medication status of patients within 30 days before follow-up, including hypoglycemic or lipid-lowering drugs.

Physical examination:

Measure the weight, height, body mass index (BMI), body fat, waist circumference, hip circumference, and upper arm circumference of all subjects. Before measuring height and weight, and visceral fat, subjects remove heavy clothing and shoes and stand. Use V-body HBF-371 (OMRON, Kyoto, Japan) to measure body fat and visceral fat. The calculation method for BMI is weight (kg) divided by the square of height (m^2^). When measuring waist circumference, hip circumference, and upper arm circumference, subjects stand with their arms naturally hanging down. The waist circumference is measured at the midpoint circumference of the line connecting the lower rib edge and the anterior iliac spine, the hip circumference is measured at the maximum circumference of the buttocks, and the upper arm circumference is measured at the midpoint circumference of the line connecting the acromion and the olecranon. Use a soft tape to measure closely to the corresponding position on the skin, accurate to 0.1cm.

Blood pressure and ankle-brachial index: Before measuring blood pressure, subjects are asked to urinate and rest for at least 30 minutes. Sit and use a professional portable blood pressure monitor (OMRON, Kyoto, Japan) to measure blood pressure and pulse of the right arm three times, with an interval of 30 seconds. Lie on your back and use VP-1000 (OMRON, Kyoto, Japan) to measure blood pressure of the four limbs three times, with an interval of 30 seconds. Calculate the average value of the three measurements for analysis. The calculation method for ankle-brachial index (ABI) is the systolic pressure of the ankle joint divided by the systolic pressure of the brachial artery.

Auxiliary examination:

Electrocardiogram examination: Electrocardiogram examination is operated by experienced cardiovascular physicians from Suzhou Hospital of Traditional Chinese Medicine. Before the electrocardiogram examination, subjects are asked to urinate and rest for at least 5 minutes. Lie on your back, and record the 12-lead electrocardiogram at a speed of 25mm/s and 1mv/cm with standard equipment. If the depth of the S wave in V1 + the highest R wave height in V5 or V6 > 3.5mv, it suggests LVH.

Ultrasonic examination: Echocardiography is performed according to the guidelines recommended by the American Society of Echocardiography (29). Obtain parasternal long-axis and short-axis, apical four-chamber, subcostal four-chamber, and other sections, measure cardiac ultrasound data, including left atrial internal diameter (LAD), left ventricular end-diastolic diameter (LVEDD), left ventricular end-systolic diameter (LVESD), interventricular septal thickness (IVST), left ventricular posterior wall thickness (LVPW), the ratio of mitral valve flow E peak velocity to the average of the lateral and septal early diastolic velocities at the mitral annulus (E/e’), etc., and measure left ventricular ejection fraction (LVEF) by the biplane Simpson method.

Abdominal ultrasound: After fasting for 12 hours, lie on your back and record the parameters of liver ultrasound according to the guidelines of the Hepatology Branch of the Chinese Medical Association, to assess the condition of liver fat metamorphosis (30).

Carotid ultrasound: Lie on your back with the neck elevated to recline the head, and slightly turn the head to one side to fully expose the neck, continuously obtain the cross-section and longitudinal section of the common carotid artery, internal and external carotid artery bifurcation, internal carotid artery, and external carotid artery branches, and measure the intima-media thickness (IMT) of the carotid artery. IMT is defined as the distance between the inner lumen and the interface between the inner and outer membranes of the vascular wall. IMT≥1.5mm or a local change that is thicker than the adjacent IMT by >0.5mm or 50% and protrudes into the lumen is considered a carotid plaque. If a plaque is detected, the location, number, size, shape, and echo characteristics of the carotid plaque need to be measured.

Pulse wave velocity examination: Pulse wave velocity examination (PWV) is performed by experienced cardiovascular physicians from Suzhou Hospital of Traditional Chinese Medicine. Subjects need to rest for at least 5 minutes before the examination, maintain a calm state, and lie on their back with the neck elevated to recline the head, and slightly turn the head to one side. Place pressure sensors at the most obvious places of carotid and femoral artery pulsation, obtain arterial pulse waveforms, record pulse wave transmission time, and calculate the carotid-femoral pulse wave velocity (cfPWV) of the subjects. Place sensors at the measurement sites of the ankle artery and brachial artery, and calculate the brachial-ankle pulse wave velocity (baPWV). Record the pulse wave transmission time, waveform characteristics, and heart rate and blood pressure of the subjects during the measurement to comprehensively assess arterial elasticity and its changes.

Chest CT examination: Use 64-slice spiral CT to scan the heart, performed by experienced radiology professionals. Score the coronary artery calcification by Agatston scoring (31), that is, the total calcium score = ∑(calcification density score) × calcification area; the total coronary artery calcification score is the sum of the calcification scores of the left main trunk, anterior descending branch, circumflex branch, and right coronary artery branches.

Retinal microvascular assessment:

Retinal microvascular assessment was conducted by experienced ophthalmologists at Suzhou Hospital of Traditional Chinese Medicine. Using a 45°color fundus camera, color fundus photographs of both eyes were taken with the optic disc and macula as the centers, and data from the right eye were selected for analysis. The IVAN computer-assisted program (University of Wisconsin, USA) was used to measure the diameters of 6 major branches of retinal arterioles and venules within a range of 0.5 to 1.0 disc diameters from the optic disc margin. The central retinal artery equivalent (CRAE) and central retinal vein equivalent (CRVE) were calculated using the Parr-Hubbard formula (32). The arteriole-to-venule ratio (AVR) was then determined as AVR = CRAE/CRVE.

Biochemical indicators examination:

Subjects fast for >8 hours and collect elbow venous blood and urine samples the next morning. Detect fasting biochemical indicators: blood glucose (GLU), glycated hemoglobin (HbA1c), fasting insulin (fast insulin, FIN) level, and calculate the insulin secretion index and insulin resistance index. The calculation method for the insulin secretion index is fasting insulin divided by fasting blood glucose concentration. The calculation method for insulin resistance index (Homeostatic Model Assessment of Insulin Resistance, HOMA-IR) is fasting blood glucose multiplied by fasting insulin divided by 22.5. The following indicators were also measured: aspartate aminotransferase (AST), alanine aminotransferase (ALT), albumin (ALB), γ-glutamyl transferase (γ-GGT), total bilirubin (TBi), direct bilirubin (DBi), indirect bilirubin (IBi), blood creatinine (SCr), uric acid (UA), glomerular filtration rate (eGFR), triglycerides (TG), cholesterol (CHOL), high-density lipoprotein cholesterol (HDL-C), low-density lipoprotein cholesterol (LDL-C), apolipoprotein A1 (ApoA1), apolipoprotein B (ApoB), lipoprotein (a) [LP(a)], thyroid-stimulating hormone, free thyroxine (FT4), free triiodothyronine (FT3), homocysteine (Hcy), C-reactive protein (CRP), interleukin-6 (IL-6), interleukin-1β (IL-1β), tumor necrosis factor-α (TNF-α) and lipopolysaccharide (LPS).

Tongue coating microbiota collection:

Subjects fast for >8 hours and collect tongue coating samples the next morning. Roll the swab from the root to the tip of the tongue for 1 time, place the swab into an EP tube containing 1 mL of phosphate-buffered saline (PBS), gently stir the swab to wash off the tongue coating microbiota. Repeat the above steps with a new swab twice to ensure sufficient collection of tongue coating samples. After collection, centrifuge (10000×g, 4°C, 15 min), gently absorb and discard the supernatant, take the sediment as the sample, freeze with liquid nitrogen, and store at -80°C for analysis.

### Outcome and follow-up

The primary outcome of this study is the first occurrence of coronary heart disease and stroke, with the earlier occurrence as the standard, and the secondary outcome is subclinical target organ damage. Coronary heart disease includes other coronary heart diseases (ICD-10 codes I23-I25), angina pectoris (I20), myocardial infarction (I21, I22). Cerebral stroke includes hemorrhagic stroke (I60-I62) and ischemic stroke (I63). Other unspecified strokes are classified as I64. Secondary outcomes are the first occurrence of subclinical target organ damage. All subjects will be followed up once every 2 years after the completion of the baseline survey. During the follow-up period, the occurrence of events will be confirmed with the same diagnostic criteria as the baseline assessment to ensure the reliability of the data. In the case of death, the death of the subject will be confirmed by the death certificate issued by the local civil registry office or community health center.

### Definition of subclinical target organ damage

Subclinical target organ damage includes cardiac, renal, vascular, and microvascular damage (33). Among them, cardiac damage includes LVH (left ventricular mass index (LVMI) > 110g/m^2^ for men and LVMI > 90g/m^2^ for women); renal damage includes MAU (ACR >30mg/g) and chronic kidney disease (CKD, eGFR <60ml/min/1.73 m^2^); vascular damage includes peripheral arterial disease (PAD, ABI <0.9), vascular stenosis (carotid or femoral artery stenosis >50%), increased carotid intima-media thickness (carotid IMT >0.9mm), arterial stiffness (AS, cf-PWV>10 m/s or ba-PWV>1400cm/s); microvascular damage includes retinopathy (retinal AVR<0.5).

### Statistics

The sample size of this study is based on the primary endpoint, which is the time to the first occurrence of coronary heart disease or stroke. Previous studies have shown that patients with cardiovascular metabolic risk factors have an increased risk of CVD compared to those without risk factors, with a hazard ratio (HR) of 1.82 (34). We set the alpha value to 0.05 and the statistical power to 80% (β= 0.20). According to the sample size calculation formula, approximately 4340 participants are needed. We expect that 10% to 15% of participants will be lost to follow-up during the follow-up process, so the final study will recruit 5000 to 6000 participants.

In prospective cohort studies, data missing and follow-up loss are common problems. We will first conduct a descriptive analysis of data missing to determine whether it is randomly missing or partially randomly missing. For randomly missing cases, only samples with complete data will be analyzed. For non-completely random missing data, multiple imputation techniques will be used to generate multiple alternative datasets to ensure the robustness of the analysis.

Random effects models will be used to deal with repeated measurement data iterated through Markov Chain Monte Carlo methods, and model the long-term trajectories of individuals and groups. Elastic net regression, random forests, tree-based methods, and support vector machines, and other machine learning methods will be used to explore the relationship between variables and outcomes, to deal with high-dimensional data and complex interactions between variables, and to find variables highly related to the risk of CVD occurrence. These models will be evaluated through cross-validation to ensure reasonable allocation of validation and training sets and to avoid overfitting and other issues. Standard random effects models combined with structural equation models will be used to explore potential causal pathways. Structural equation models can comprehensively analyze the impact of multiple variables on predictive variables, providing more comprehensive model validation. For the main outcome, we will further explore the potential mediating effect of tongue coating microbiota in the occurrence and development of CVD through structural equation models. To evaluate the predictive performance of the model, standardized indicators such as accuracy, precision, recall, F1 value, and area under the ROC curve will be used for assessment.

All statistical analyses will be completed in R software, using its related packages such as lme4 for random effects models, glmnet for elastic net regression, randomForest for random forest analysis, lavaan for structural equation model analysis. Machine learning analysis will also be implemented in R.

## Discussion

The pathogenesis of CVD is complex, involving the combined action of demographic, environmental, lifestyle, genetic, and immune factors (35, 36). This study is based on the concept of cardiovascular 1.5-level prevention proposed by us, focusing on subclinical target organ damage, which makes up for the shortcomings of traditional risk assessment tools in the early pathological changes. As a prospective cohort study conducted in Suzhou, China, the “Suzhou Cardiometabolic Health (SCH) Study” is committed to revealing the unique role of oral (tongue coating) microbiota and multiple metabolic factors in cardiovascular risk assessment, promoting the implementation of more precise 1.5-level prevention strategies. Suzhou is located in the Yangtze River Delta economic zone, with a unique population composition and dietary pattern, providing an important background for the study of cardiovascular metabolic health. Through systematic follow-up of individuals of different age groups and metabolic states in this region, the SCH study will provide a scientific basis for future multi-dimensional CVD risk assessment tools and personalized health management. 1.5-level prevention reflects the concept of “prevention before disease”, aiming to reduce the occurrence of CVD and subclinical target organ damage through earlier identification and intervention, and promoting personalized precision medicine.

This study introduces tongue coating microbiota characteristics as metabolic markers for the first time, exploring their potential role in CVD risk assessment. In recent years, more and more evidence has shown that oral microbiota plays an important role in metabolic cardiovascular diseases, especially its complex interactions with inflammation, immune regulation, and endothelial dysfunction (21, 37, 38). Our team’s previous cohort study discovered and validated that the increased abundance of *Fusobacterium nucleatum* in the oral cavity is one of the notable characteristics of patients with diabetic coronary heart disease. Moreover, the oral-gut microbial transmission serves as a crucial intermediary mechanism through which diabetes affects myocardial ischemia-reperfusion injury, laying the groundwork for further exploration of the regulatory role of oral microbiota in cardiovascular and metabolic health (37). However, high-throughput sequencing data is insufficient, the field lacks large-scale, long-term clinical cohorts that integrate diverse metabolic factors and oral microbiota. Our research should focus on the diversity, composition, functional potential of tongue coating microbiota, coupled with advanced approaches such as machine learning model construction (39, 40). Species such as *Fusobacterium nucleatum* and members of the “red complex” (*Porphyromonas gingivalis*, *Tannerella forsythia*, and *Treponema denticola*) have been implicated in systemic inflammation and atherosclerosis, underscoring their potential role as biomarkers or therapeutic targets in cardiovascular health (41, 42).

At the same time, this study adopts a prospective, large-scale cohort design, covering comprehensive data collection from lifestyle to metabolic indicators, including subjects’ diet, exercise, sleep, smoking, and drinking factors. By detailed recording and analysis of these lifestyle factors, this study can comprehensively assess the risk of cardiovascular diseases from multiple dimensions, providing lifestyle suggestions for clinicians and patients, promoting interdisciplinary cooperation between cardiovascular science and endocrinology, nutrition, and other fields, to meet global health challenges.

The main advantages of this study are the diversity of the study population, the application of high-throughput tongue coating microbiota sequencing technology, and advanced statistical schemes. The study not only includes laboratory indicators such as sugar and lipid metabolism but also pays special attention to young people with unhealthy lifestyles, providing a broader perspective for the identification of cardiovascular disease risk. The introduction of tongue coating microbiota as a new metabolic marker not only helps in the early identification of cardiovascular risk but also provides a modern scientific basis for traditional Chinese medicine tongue diagnosis theory. Traditional Chinese medicine theory believes that tongue coating reflects the internal state of the human body and is closely related to metabolic disorders such as phlegm and dampness. Research shows that this macro concept may have specific scientific connotations. For example, tongue coating is highly related to microbiota, and the disorder of tongue coating microbiota can promote the progression of coronary heart disease (43, 44). This study explores this concept and provides modern scientific evidence for the traditional Chinese medicine theory of tongue diagnosis.

This study also has some limitations. First, this study is a single-center cohort study, which may affect the generalizability of the results. Second, the sequencing and analysis of tongue coating microbiota depend on highly specialized equipment and technology, which may limit its promotion in a larger population. Finally, the longer follow-up period may lead to some participants being lost to follow-up, thereby affecting the completeness and robustness of the final data and results.

In the future, we will focus on conducting multi-center studies and aim to expand the applicability of tongue coating microbiota characteristics across different regions and populations. Second, exploring how to incorporate the strategy of cardiovascular 1.5-level prevention into daily clinical practice, especially in health management and risk assessment, to ensure that high-risk individuals can receive intervention as early as possible is also an important topic for the future. In addition, future research will explore the potential mechanisms of tongue coating microbiota in the occurrence of CVD, especially its interactions with other metabolic markers. By early identification and intervention of pre-disease damage in cardiovascular diseases, this study hopes to provide high-quality evidence for reducing the incidence of CVD and improving the overall health level of the population.

## Conclusion

In summary, this study proposes an integrated multi-dimensional factor CVD risk assessment strategy by combining oral (tongue coating) microbiota, a new type of metabolic marker, with traditional metabolic factors. This study aims to verify the feasibility and effectiveness of this new method in identifying subclinical target organ damage and CVD risk stratification, striving to improve the early identification and precise prevention level of CVD, thereby providing a scientific basis for cardiovascular 1.5-level prevention.
