# Supplementary material for: Advancing early detection of organ damage and cardiovascular risk prevention: the Suzhou cardiometabolic health study protocol - exploring the role of oral microbiome and metabolic profiling in risk stratification
Source: Front Endocrinol (Lausanne). 2025 Feb 12;16:1522756. doi: 10.3389/fendo.2025.1522756 (PMC11860102; doi:10.3389/fendo.2025.1522756)
Supplement: Supplementary file 1 [file Table1.docx]

**Supplemental File**

**Tables:**

**1. Table S1. International Physical Activity Questionnaire Short Form**

**2. Table S2. Food Frequency Questionnaire**

**Table S1 International Physical Activity Questionnaire Short Form**

1. In the last 7 days, on how many days did you engage in vigorous physical activities, such as carrying heavy loads, digging, aerobics, or fast cycling?

days per week □ No vigorous physical activity → skip to Question 3

2. On a typical day of such vigorous activities, how much time did you spend on them?

hours minutes per day □ Don’t know or unsure

3. In the last 7 days, on how many days did you engage in moderate physical activities, such as carrying light loads, cycling at a regular pace, or playing doubles tennis? (Do not include walking.)

days per week □ No moderate physical activity → skip to Question 5

4. On a typical day of such moderate activities, how much time did you spend on them?

hours minutes per day □ Don’t know or unsure

5. In the last 7 days, on how many days did you walk for at least 10 minutes at a time?

days per week □ No walking → skip to Question 7

6. On a typical day of walking, how much time did you spend walking?

hours minutes per day □ Don’t know or unsure

7. On weekdays in the last 7 days, how much time did you spend sitting?

hours minutes per day □ Don’t know or unsure

**Table S2 Food Frequency Questionnaire**

In the past 12 months, how often did you consume the following foods? Please check the appropriate frequency and select the usual cooking method.

| **Food Category** | **Daily** | **4-6days/**  **week** | **1-3 days/**  **week** | **Monthly** | **Never/**  **Rarely** | **Common Cooking Method** | |
| --- | --- | --- | --- | --- | --- | --- | --- |
| Rice | □ | □ | □ | □ | □ | —— | |
| Noodles | □ | □ | □ | □ | □ | —— | |
| Wheat products (e.g., steamed buns, bread) | □ | □ | □ | □ | □ | —— | |
| Red meat (e.g., pork, beef) | □ | □ | □ | □ | □ | □Steamed □Boiled  □Pan-fried □Stir-fried | □Grilled □Braised  □Deep-fried □Other |
| Poultry (e.g., chicken, duck) | □ | □ | □ | □ | □ | □Steamed □Boiled  □Pan-fried □Stir-fried | □Grilled □Braised  □Deep-fried □Other |
| Freshwater fish/seafood | □ | □ | □ | □ | □ | □Steamed □Boiled  □Pan-fried □Stir-fried | □Grilled □Braised  □Deep-fried □Other |
| Soy products (e.g.tofu, soy milk) | □ | □ | □ | □ | □ | □Steamed □Boiled  □Pan-fried □Stir-fried | □Grilled □Braised  □Deep-fried □Other |
| Fresh fruits | □ | □ | □ | □ | □ | —— | |
| Fresh vegetables | □ | □ | □ | □ | □ | □Steamed □Boiled  □Pan-fried □Stir-fried | □Grilled □Braised  □Deep-fried □Other |
| Vegetable oil (e.g. olive oil, rapeseed oil) | □ | □ | □ | □ | □ | —— | |
| Dairy products (e.g. milk, yogurt) | □ | □ | □ | □ | □ | —— | |
| Nuts (e.g. walnuts, peanuts) | □ | □ | □ | □ | □ | —— | |
| Tea (e.g. green tea, black tea) | □ | □ | □ | □ | □ | —— | |
